# Initial findings from the implementation of a community-based sentinel surveillance system to assess the health effects of climate change in Alaska

**DOI:** 10.3402/ijch.v72i0.21405

**Published:** 2013-08-05

**Authors:** David L. Driscoll, Tenaya Sunbury, Janet Johnston, Sue Renes

**Affiliations:** 1 Institute for Circumpolar Health Studies, University of Alaska, Anchorage, AK, USA; 2 School of Education, University of Alaska, Fairbanks, AK, USA

**Keywords:** community-based, surveillance, adaptation, climate change, Alaska

## Abstract

**Background:**

This report describes the results of a study to determine whether a community-based sentinel surveillance system can be developed and implemented to assess the health effects of climate change, and to contribute to local discussions to mitigate these health effects. The purpose of this report is to describe the process and outcomes of this innovative approach to identifying priority areas for adaptation investment. This report can be used to assist local, state and federal governments in determining how to develop actions and policies to promote adaptation to climate change.

**Objective:**

To evaluate the health effects of climate change in rural Alaska.

**Design:**

We conducted an iterative and participatory process to develop metrics, an instrument and a protocol to collect sentinel surveillance data on the health effects of climate change in 3 ecologically distinct regions of the state.

**Results:**

We collected surveillance data from 91 study participants over the course of 12 months. These data were analyzed and categorized by frequency and association between specific health outcomes or health-related factors (such as food security) and reported exposure to environmental effects of climate change. We found significant associations between several health outcomes and health outcome mediators and reported exposures. We presented these data to study participants in community settings and moderated discussions of likely causal factors for these measured associations, and helped community residents to identify specific adaption measures to mitigate those health effects.

**Conclusions:**

We conclude that community-based sentinel surveillance is an effective method for assessing health outcomes from exposure to environmental effects of climate change, and informing climate change health adaptation planning in Alaskan communities. We contend that it would be effective in other regions of the nation as well.

Climate records in Alaska indicate that the average temperature in portions of the state has increased by 7°F since the 1950s. There have been a number of documented changes related to this warming trend, including the degradation of permafrost, loss of sea ice, and warming and acidification of seawater (1). Residents of the circumpolar north have long provided anecdotal reports of unusual shifts in the behavior and health of fish and game, subsidence of ground and surface water levels, and increasingly extreme local weather patterns with attendant health consequences. Inuit residents of the Rigolet, Nunatsiavut, Canada, for example, perceive associations between a changing climate and many physical and mental health outcomes in their community (2).

Variations in regional climactic conditions represent just one component of a framework for conceptualizing and mitigating the health effects of climate change. Moderating influences associated with geographical and socio-economic conditions represent yet another important consideration (3). Some communities will be more vulnerable to the health effects of climate change than others due to an ability to respond to these challenges through government action, community awareness, and the ability to facilitate community response (4). Other moderating influences include population growth and demographic change (e.g. seasonal employment/tourism), level of economic and technological development, local knowledge and resilience, pre-existing health status, the quality and availability of health care, and public health infrastructure (5, 6).

We collected data on local environmental events likely to be associated with climate change, the health impacts of these events, and worked with communities to identify high-priority and actionable adaptation strategies to mitigate those effects on their health. This article describes the process by which our study methodology was developed, evaluates the effectiveness of that methodology and provides the adaptation measures recommended by residents in each of the 3 regions of Alaska to mitigate the health effects of climate change in their communities.

## Methods

### Study sites

**Fig. 1:**
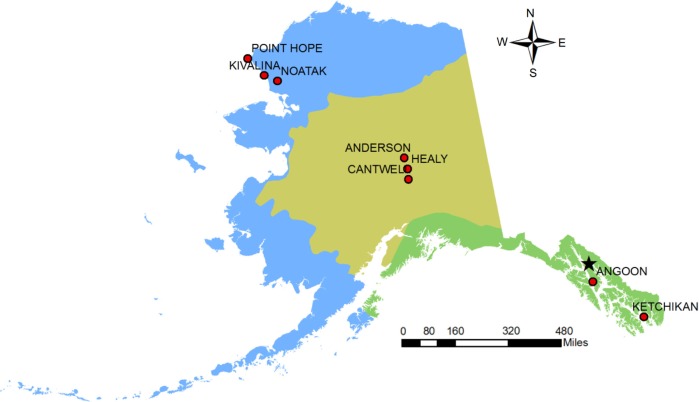
Study sites.

Surveillance data were collected by residents of the city and village of Ketchikan and Angoon in the Southeast region of the state, the villages of Healy, Anderson, and Cantwell in Interior, and the villages of Point Hope, Kivalina, and Noatak in Northwestern, Alaska (Fig. 1). These 8 population centres provide a representative cross-sample of the state's population across 3 ecologically distinct regions – the Southeast, Interior, and Northwest:

- In 2010, Ketchikan and Angoon had 8,509 residents living in 1,998 family households and 1,428 non-family households. Non-family households are composed of people who live alone or non-relatives living together, including unmarried partners or roommates. The racial makeup of the population centres was approximately 58.00% White, 0.79% Black or African American, 19.90% Native American, 10.20% Asian, 0.27% Pacific Islander, 0.68% from other races, and 10.17% from 2 or more races.
- In 2010, the villages of Healy, Anderson, and Cantwell had 1,486 residents living in 377 family households and 251 non-family households. The racial makeup of the 3 villages was 88.76% White, 0.61% Black or African American, 4.17% Native American, 1.21% Asian, 0.07% Pacific Islander, 0.47% from other races, and 4.71% from 2 or more races.
- In 2010, the villages of Point Hope, Kivalina, and Noatak had 1,562 residents living in 315 family households and 70 non-family households. The racial makeup of the 3 villages was 3.84% White, 0.32% Black or African American, 92.83% Native American, 0.06% from other races, and 2.94% from 2 or more races.

### Study design

**Fig. 2:**
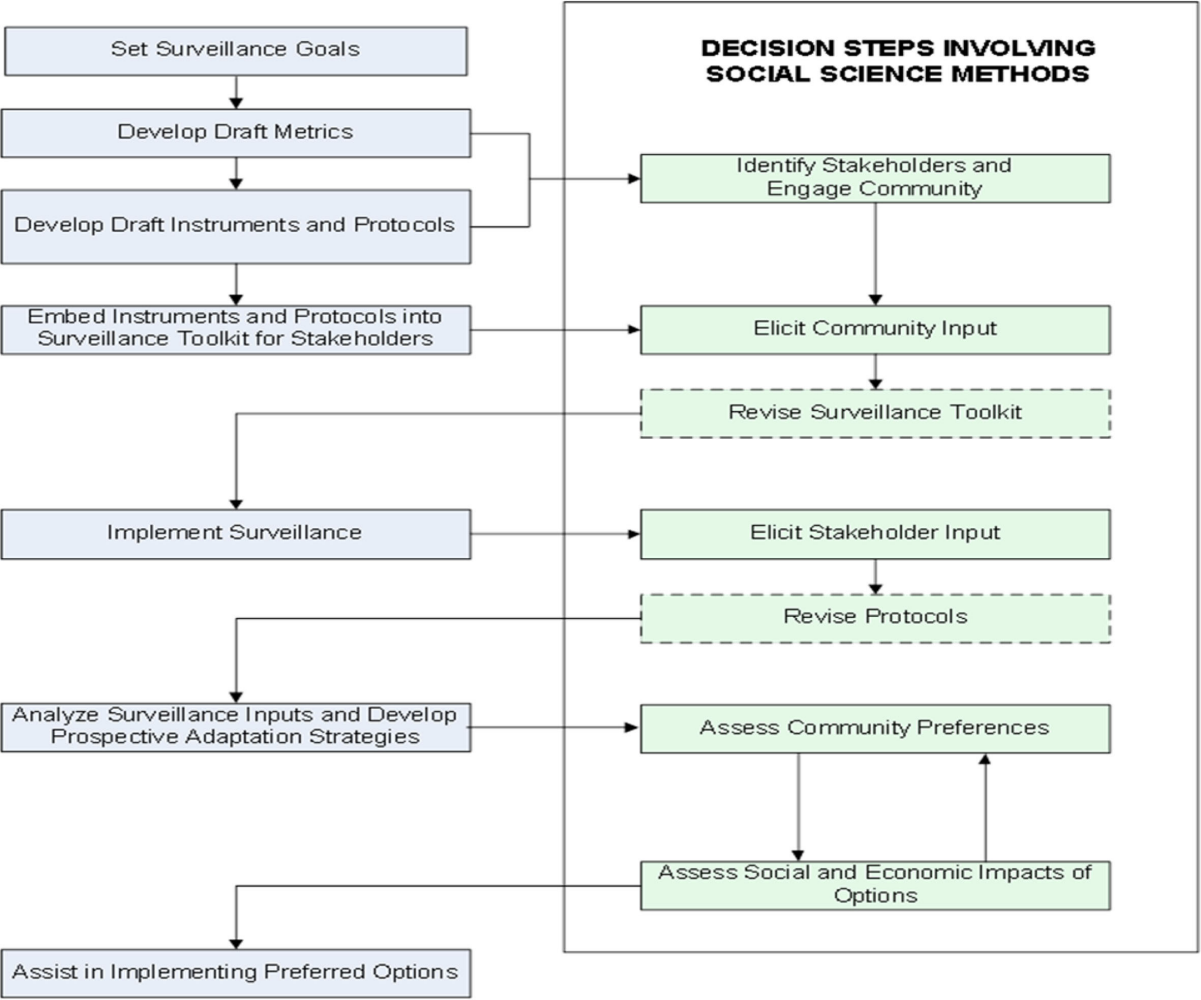
Study design.

Our interdisciplinary study design integrated public health and social scientific approaches to develop and conduct a community-based sentinel surveillance system for the environmental effects of climate change and their associated health impacts. Community member participation was an important part of the study design, both for providing surveillance data and following data collection to inform community preferences regarding prospective adaptation strategies to mitigate those health effects in the future (Fig. 2).

### Surveillance goals and metrics

A colloquium of government and academic stakeholders, including Health Canada, Trent University in Ontario, Canada, the US Centers for Disease Control and Prevention, the US Environmental Protection Agency, the Alaska Native Tribal Health Consortium, and the University of Alaska system recommended that the project investigators categorize prospective effects of climate change into basic categories by which residents of rural and isolated communities experience their environment: water, atmosphere, land, etc. They further recommended that project investigators be aware of the effects of seasonality on all these elements, and ensure that data are collected year-round. The list of categories and attendant surveillance goals is presented in Table I.

**Table I:** Surveillance goals

Surveillance elements	Observations
Atmosphere	Changes in: weather extremes/variability (increase); weather predictability (increase); particulate matter (increase) from fires; allergen concentration (increase); UV–B radiation (increase), temperature (increase)
Water	Changes in: sea ice; precipitation (rain, snow) (quantity and quality); river and lake systems (flow, sediment, contaminants, temperature); freeze-up timing (later); break-up timing (earlier); ocean acidification (increase acidification or lower pH) Storm surges (loss of protection from sea ice)
Land	Changes in built environment (road networks, sanitation infrastructure, wastewater and solid waste systems), permafrost active layer (change) Coastal areas Retreating glaciers: erosion, land uplift
Biology/ecosystems	Invasive flora and fauna species Changes in priority flora and fauna species (numbers, trends, and distribution)

Social	Cultural issues (e.g. changes in human and industrial activity due to ice-free ocean passage, traditional food gathering, preparation, storage, and consumption) Acute and chronic mental health events? Public health workers and/or emergency responders (what about them?) Causes of morbidity and mortality

We used the United States CDC National Health and Nutrition Survey (NHANES) and Behavioral Risk Factor Surveillance Survey (BRFSS) as surveillance survey design templates (7, 8). These large-scale, repeated surveys ask participants to self-report on a variety of structured health outcomes and behaviors. Using a similar model, we prepared a spreadsheet utilizing colloquium climate change topics and started with major headings of: research question number, research purpose, prospective survey questions (as primary data collection), secondary data source (for the evaluation of health and ecosystem data) and any additional comments for the interview question. We populated an initial survey instrument featuring at least one survey question for each research objective in the spreadsheet.

**Fig. 3:**
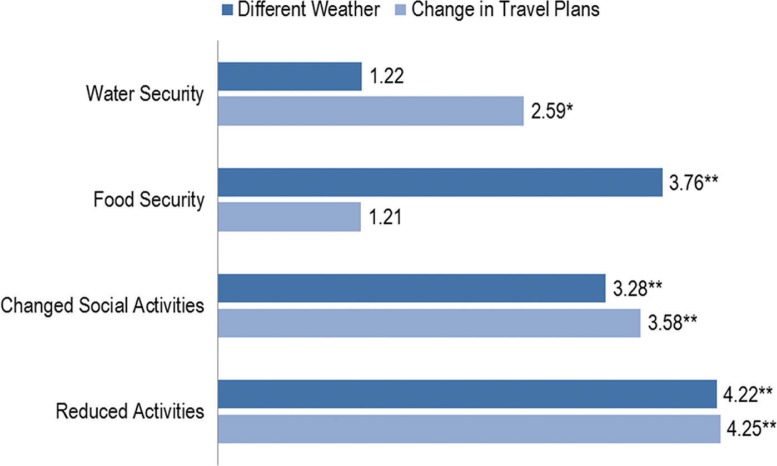
Health outcome mediators associated with exposures.

The initial instrument consisted of 31 items with multiple response options. We pre-tested these metrics in a small convenience sample of long-term residents of the circumpolar north, including Canadian researchers and environmental coordinators in Alaskan villages. Some of our pre-test participants described the questions as too long and detailed. Subsequent reviews of the circumpolar health literature suggested that some of the survey questions may have been methodologically challenging in rural and isolated villages (9).

Alaska has one of the highest rates of computer ownership and internet access in the United States (10), and the initial protocol specified a web-based survey. After discussions with community organizers and key informants, however, 2 other data collection modes were added in the hope of increasing response rates. These included telephone surveys and a mail-in option. Program investigators established a toll-free 1-855 telephone number for residents who preferred to communicate via phone for a variety of reasons (e.g. oral cultural tradition, insecure in spelling/writing skills). Self-addressed, stamped envelopes and 12 survey forms were printed and included in each participant's toolkit along with waterproof pocket notepads to facilitate day-to-day collection of participant observations.

The final iteration of the sentinel surveillance survey contains 5 thematic parts with a total of 34 items (33 items that consist of a 4- or 2-point structured response scale). The first section consists of community observations on local weather (12 items), the second asks respondents to report on community observations on hunting and harvesting (8 items), the third covers community observations on food and water safety (7 items), the fourth covers community observations on general health and air quality (6 items), and the last is an open-ended text section that allows respondents to report on any additional community observations. This last field allowed participants to provide information on moderating factors, or population-level characteristics and non-climate factors that vary the health risk/susceptibility of populations. With these revisions, the monthly survey required approximately 30–40 min to complete. An incentive of $20 per completed monthly survey was deemed appropriate for this investment in time, and was provided to all participants. The instrument and protocol were reviewed and approved by the UAA Institutional Review Board.

Table II provides a summary of the decisions made during the instrument development process.

**Table II:** Initial to final survey design

Surveillance survey characteristics	Initial design	Final design
Data collection	Quantitative data only using likert scales	Qualitative and quantitative data using likert scales and one open-ended text field for additional comments
Data collection mode	Web only	Mail-in, toll-free telephone number, and web
Survey items	Detailed list of hunted animals/fish categorized by animal size and list of harvestable plants	Reduced to 2 survey items asking residents to report on any changes to hunting/harvesting
Survey style	None	Thematic sections and likert scales were highlighted and directions were provided at regular intervals
Toolkit materials	None	Waterproof and re-useable material and self-addressed, stamped envelopes

### Study implementation

The final study instrument and protocol were implemented with repeated submissions of structured observations of the same metrics over the course of 12 months beginning in the spring of 2011. We were well aware of the experiences that indigenous people have had with researchers, including climate change research (11), and in the first month of the study we spent several days in each study community meeting with participants in one-on-one and group settings to discuss the study objectives and protocols, and to provide training in completing and returning the instruments. None of the study participants requested revisions to the study instrument and protocol in these discussions.

### Data analysis

We identified high-frequency health outcomes and risk factors based on the responses from individual observers based in 8 study communities in 3 distinct geographic regions. These health-related observations were ranked by frequency overall and within the 3 regions.

We examined associations between the following 2 exposure variables and a variety of environment-related health risk factors and health outcomes:

- Exposure Q1: “In the past 30 days, how often has the weather seemed very different from what you (or people you know in the community) expect this time of year?” Responses were dichotomized to “never” and “at least one time or more”.
- Exposure Q2: “In the past 30 days, how often have you (or people you know in the community) changed when and how you travel as a result of unusual weather?” Responses were dichotomized to “never” and “at least one time or more”

We calculated odds ratios (ORs) and 95% confidence intervals (CIs) for the likelihood of each of the health risk factors or outcomes occurring in communities experiencing the exposure one or more times in the past 30 days as compared to the likelihood of that risk factor or outcome occurring in a community without the exposure during the past 30 days. Using SAS version 9.2, PROC GENMOD with a logit link, we conducted a generalized estimating equation (GEE) analysis to account for repeated measures from individual observers nested in communities (12, 13).

## Results

### Surveillance participants

A total of 91 individuals across the 3 study regions provided at least one monthly surveillance instrument over the 12 months of primary data collection. These participants were selected on the basis of 3 eligibility criteria: age 18 or older, a resident of the study region for a minimum of 10 years, and capable of understanding and completing surveys written in English. Although age was not a selection criterion, the median age of study participants was 56 years. On a monthly basis, as many as 66, and as few as 44, study participants returned surveillance instruments. Due to the shifting, seasonal nature of residence patterns in the northwest, study participants continued to participate in the study while residing in neighbouring villages in each study region if they met the inclusion criteria and could provide observations of events taking place in the study village. Table III provides a detailed breakdown of the number of participants providing instruments per month, per community.

### Surveillance observations

The highest frequency observations of health outcomes and risk factors potentially associated with environmental changes were water and food security, followed by various forms of unintentional injury, and paralytic shellfish poisoning. Many other health outcomes or risks were identified in specific communities. Table IV presents the rank-order of the 5 highest frequently reported health-related observations state-wide by region and across the study population.

**Table III:** Study participants by month and region

Month	Southeast	Northwest	Interior
April	23	29	14
May	16	17	11
June	13	17	14
July	13	19	14
August	17	19	17
September	18	18	9
October	15	18	11
November	16	20	12
December	12	19	15
January	16	19	15
February	16	17	16
March	16	15	15

**Table IV:** Health-related observations

	Rank				
	
Region	1	2	3	4	5
Northwest	Water security	Food security	Cold-related injuries and fatalities	Allergic asthma	Paralytic shellfish poisoning
Interior	Food security	Cold-related injuries and fatalities	Allergic asthma	Water security	Paralytic shellfish poisoning
Southeast	Water security	Allergic asthma	Food security	Cold-related injuries and fatalities	Paralytic shellfish poisoning
*Combined*	*Water security*	*Food security*	*Allergic asthma*	*Cold-related injuries and fatalities*	*Paralytic shellfish poisoning*

### Health outcomes associated with reported exposure changes

We examined the relationship between 2 exposures (i.e. unusual changes in weather and travel) in each monthly survey and health outcomes, such as allergic asthma, paralytic shellfish poisoning (PSP), hypothermia, frostbite, unintentional injury and perceptions of overall community health (Table V). Reported results were significant at a p-value less than 0.05 unless noted. Communities who reported any “unusual” changes in weather during the previous 30 days were 3.62 (95% CI 1.13–11.65) times more likely to report unintentional injuries and 1.83 (95% CI 1.00–3.36) times more likely to report “acceptable or poor” (vs. “excellent or very good”) overall individual and community health than communities who did not report any weather changes adjusting for sex and race.

Communities who reported any changes in travel plans during the previous 30 days were 4.70 (95% CI: 1.56–14.15) times more likely to report hypothermia, 3.67 (95% CI: 1.95–6.92) times more likely to report frostbite (clustered by region), 4.72 (95% CI: 2.49–8.94) times more likely to report an unintentional injury and 1.59 (95% CI: 0.99–2.58, p <0.06) times more likely to report “acceptable or poor” individual and community health than communities who did not report any weather changes adjusting for sex and race. There were an insufficient number of PSP observations to model these associations (see Fig. 3).

### Health outcome mediators associated with reported exposure changes

We also examined the relationship between the same 2 exposures in each monthly survey response and mediators of health outcomes, such as reduced water security, reduced food security, changes in social activities, and changes in work or other activities. Reported results were significant at a p-value <0.05. Communities who reported any changes in travel plans during the previous 30 days were 3.76 (95% CI: 1.13–11.65) times more likely to report reduced food security, 3.28 (95% CI: 1.67–6.46) times more likely to report changes in social activities, and 4.22 (95% CI: 2.17–8.19) times more likely to report changes in work or other activities than communities who did not report any weather changes adjusting for sex and race.

Communities who reported any changes in travel plans during the previous 30 days were 2.59 (95% CI: 1.26–5.36) times more likely to report reduced water security, 3.58 (95% CI: 2.01–6.39) times more likely to report changes in social activities and 4.25 (95% CI: 2.65–6.80) times more likely to report changes in work or other activities than communities who did not report any travel changes adjusting for sex and race.

**Table V:** Health outcomes associated with exposures ORs^a^ clustered by community

Outcome	Exposure 1: Unusual weather	Exposure 2: Changed travel plans
Water security	1.22	2.59*
Food security	3.76**	1.21
Allergic asthma	1.53	1.09
PSP	Cannot model	
Hypothermia	2.86	4.70**
Frostbite^b^	1.99	3.67**
Injury	3.62*	4.72**
Poorer overall health	1.83*	1.59

a Adjusted for sex and race

b clustered by region.

* p<0.05

** p<0.01.

### Community adaptation mapping

Following collection and analysis of the survey data from each study community, project investigators conducted a second round of site visits to present surveillance findings and lead a community mapping exercise to identify and prioritize community preferences for adaptation measures. Surveillance findings were presented to study participants, and any other interested members of the study community, in the form of a poster-enhanced group discussion. These informal discussions allowed study participants to voice their agreement or disagreement with the surveillance findings, to discuss associations between the primary environmental and health outcomes described in the surveillance data and to identify prospective adaptation strategies to mitigate those causal associations. These discussions incorporated a mapping exercise that sought to link those sections of the community observations posters that detailed environmental changes with those related to participant identified health outcomes.

Residents of Southeastern Alaska linked several health outcomes to local environmental changes. These include unintentional injuries which residents associated with deteriorating infrastructure from frost-heave and intense rain events, and allergies and other respiratory ailments which residents linked to increasing amounts of black mould in homes. Residents also named declining food security as a priority health problem associated with increased levels of the toxin causing paralytic shellfish poisoning. There was little regional consensus regarding the most effective solutions to these health issues. Residents of Ketchikan prioritized access to quality healthcare as the most effective adaptation strategy, whereas residents of Angoon (which unlike Ketchikan has no hospital and thus little immediate opportunity for improved access to healthcare) focused on improved health education and monitoring, both related to air quality in homes and in the natural environment.

Residents of Interior Alaska described a host of prospective causal associations linking respiratory ailments such as asthma to declining air quality associated with wind-blown dust in the spring, increasing pollen levels in fall and smoke from forest fires in summer. Some participants described problems with access to clean water, particularly among the large number of homesteaders in the region. Residents also described increasing and troubling interactions between humans and bears in the Denali region. At the time this report was being prepared, a tourist was killed by a grizzly in the Denali National Park; the first such occurrence in the park's history. Study participants felt that many of these interactions were driven by a growing number of people in the region who have little idea how to deal with bears or the scraps from hunting and fishing activities. Some participants also noted that fish and berries, food-sources for both bears and humans, seem less consistently available in the region. Prospective adaptation measures mentioned by these study participants included better monitoring and health education related to air quality concerns, educational materials for hunters on the proper disposal of the harvests and (as with the residents of Ketchikan) improved access to quality healthcare. Some participants also suggested a community laundry/shower facility in their community could reduce unintentional injuries associated with negotiating dangerous river- and stream-banks to fetch water for household use.

Residents of Northwest Alaska prioritized health issues associated with decreasing food security, and with air and water quality. Study participants in Kivalina who traditionally harvest seals and (when possible) whales described changes in the location and safety of shore-fast ice necessary for basecamps. They described several unintentional injuries associated with unexpectedly intense storms and warming and cooling trends that weaken ice or flood trails. We heard that the ice is not as thick as it used to be. Several participants described how the ice has decreased from a foot and a half to 6 inches in thickness, which inhibits travel with heavily laden snow machines and sledges. We were told by one family, “It's going to be hard to feed our kids. We have to stretch it out. Two out of the 5 Arctic Char that I cooked turned white when I cooked it and the fish crumbled and it tasted different, too. For the bearded seal hunt, we used to get 14–16 bearded seals, this year we only got 4. It was a very short year.”

Residents of Noatak who traditionally harvest caribou describe changes in the caribou migration route, as well as burgeoning numbers of predators in the region, such as bears (including an unusual number of polar bears) and wolves. Residents of both villages suggested that increased respiratory ailments were associated with higher amounts of airborne particulates (primarily dust) associated with increasingly warm and dry weather and higher wind velocities. The dry condition is seen as the cause of sickness in the children, specifically respiratory ailments, and many residents prevent their children from playing outdoors in those conditions. Some residents of Kivalina also felt that these ailments might be associated with activities at nearby Red Dog Mine, an open-pit mine that is the world's largest producer of zinc and lead. Study participants in both villages expressed concerns with erosion. The Kivalina water system was inoperative during the latter phases of this study due in part to a high particulate load in the nearby river due to heavy erosion upstream. The Noatak airstrip is under threat of closure as the Noatak River undercuts the southern edge of the property. Residents of both villages recommended that adaptation strategies include better erosion control. They also recommended that health education materials be developed and disseminated in each community, and in Kivalina this could be done through the recently hired tribal coordinator, and included in the tribal newsletter.

## Conclusion

These findings serve to illustrate several important conclusions. To begin with, the deliberate and transdisciplinary process of developing first metrics, then an instrument, and finally a primary data collection protocol in collaboration with both content-area experts and residents of rural and isolated villages in Alaska has resulted in a valid and actionable surveillance tool for use in a region of the country with few secondary data-sources. This process should prove effective in other regions of the nation as well.

Second, the collection of community-based sentinel surveillance data represents an effective strategy for assessing the nature and extent of health outcomes and health outcome mediators associated with environmental changes in communities across Alaska. The collection of such data is substantially more sensitive than more traditional passive surveillance systems and, with the inclusion of open-ended response fields, is far more flexible than many active surveillance systems requiring participants to self-disclose their health outcomes and behaviors. It is important to note that engaged and informed community residents are necessary for the successful implementation of such a sentinel-surveillance system, and thus the exposure or exposures in question must be of sufficient priority to the residents themselves that they are willing to contribute to the on-going and rigorous collection of primary data.

Thirdly, the combination of presentation/discussion of the sentinel surveillance data contributed to productive discussions of the prospective causal relationships between environmental events and the health outcomes described. For example, an explicit recognition of the association between unusual weather events and unintentional injuries provided insights into such mediating factors as unsafe ice or housing conditions, which were obvious to participants but might not have been otherwise identified by project investigators. These discussions tended to be quite far-reaching, with the scope of prospective adaptation strategies encompassing local policies and built infrastructure. This explicit recognition of linkages between individual health outcomes and ecological factors is an intuitive but unexpected benefit of this approach. The adaptation strategies that resulted from this process are both locally determined and data driven. This combination of factors would seem auspicious for the successful conduct of one or more of these adaptation strategies at the regional level.

Recommendations: We would particularly recommend efforts to disseminate data on air and water quality from existing monitoring efforts to members of these communities as a cost-effective and necessary first step toward climate change adaptation. At the very least, these should include warnings related to particulates from smoke and pollen, and perhaps include information related to monitoring for PSP outbreaks in the southeastern region of the state. Additional adaptation strategies commonly mentioned by our participants include better access to quality healthcare for unintentional injuries and other common outcomes, water systems and other infrastructure that remain safer and efficient despite growing erosion and other aspects of severe weather events, and more information on the availability of alternative foods in light of reductions in traditional subsistence food sources.

We look forward to continuing to work with these communities and describing our efforts developing and implementing these measures to mitigate the environmental challenges described herein.
